# Knowledge, training, and practice patterns in pneumatic tourniquet use among orthopedic physicians: a national cross-sectional survey

**DOI:** 10.1007/s00402-025-06176-1

**Published:** 2026-02-02

**Authors:** Mert Gündoğdu, Deniz Gülabi, Özgür Baysal

**Affiliations:** Department of Orthopedics and Traumatology, Marmara University, Istanbul, Turkey

**Keywords:** Pneumatic tourniquet, Orthopedic training, Complications

## Abstract

**Background:**

Pneumatic tourniquets are widely used in orthopedic surgery to create a bloodless operative field; however, improper use may result in skin, muscle, and nerve injuries, as well as rare but severe systemic complications. Despite their frequent use, the extent of theoretical knowledge and structured training among orthopedic surgeons remains unclear. This study aimed to evaluate knowledge levels, clinical practices, complication awareness, and training needs regarding pneumatic tourniquet use among orthopedic specialists and residents in Türkiye.

**Methods:**

This cross-sectional survey was conducted between April 1 and May 1, 2025, using an online questionnaire based on AORN 2007 pneumatic tourniquet safety guidelines. Orthopedic specialists and residents across various hospital types were invited to participate voluntarily. The questionnaire assessed demographic characteristics, training status, knowledge of safe tourniquet practices, and complication experiences. Data were analyzed using SPSS v25.0, with significance set at *p* < 0.05.

**Results:**

A total of 300 physicians participated; 45.3% were senior specialists. Although tourniquet use was frequent (48.7% in almost every surgery), only 35.7% received both theoretical and practical training. Notably, 248 participants reported that their knowledge was gained primarily through residency experience rather than structured education. Skin injuries (41.3%), muscle weakness (37.3%), and nerve injury (30.3%) were the most common complications. Knowledge gaps were prominent, particularly regarding IVRA-related systemic toxicity (24% correct), prolonged tourniquet use complications (48.3% correct), and physiological changes after deflation (68.7% correct). Experience and training level showed limited impact on overall knowledge, except for the question regarding prolonged tourniquet duration (*p* = 0.01).

**Conclusion:**

Despite widespread use, substantial deficiencies exist in theoretical knowledge and structured training on pneumatic tourniquet application. Reliance on experiential learning during residency appears insufficient and contributes to inconsistent practices and increased complication risk. Standardized, evidence-based training programs are urgently needed to improve safety and reduce variability in clinical practice.

**Supplementary Information:**

The online version contains supplementary material available at 10.1007/s00402-025-06176-1.

## Introduction

Pneumatic tourniquets are widely used in extremity surgery to provide a bloodless operative field, thereby enhancing surgical visibility, procedural efficiency, and overall operative safety [1–4]. In addition to their routine use in orthopedic procedures, tourniquets are also an essential component of intravenous regional anesthesia (IVRA), where they enable the intravenous administration of local anesthetics to achieve temporary anesthesia in the distal extremity [5].

Despite their widespread and seemingly straightforward use, pneumatic tourniquets are associated with a broad spectrum of potential complications when applied improperly. These complications include skin and soft tissue injuries, peripheral nerve damage, thermal burns, ischemia–reperfusion injury, rhabdomyolysis, thromboembolic events, pulmonary embolism, and compartment syndrome [6–9]. Therefore, determining appropriate pressure levels, not exceeding the maximum safe usage time, and correct placement of the devices are critical for proper tourniquet use [10].

Physicians are responsible for the safe use of tourniquets and for managing tourniquet-related complications [11]. However, knowledge, technical skills, and routine practices regarding tourniquet use are influenced by multiple factors, including clinical experience, formal training exposure, and institutional protocols. Although a limited number of national studies have previously assessed pneumatic tourniquet practice patterns [12, 13], these investigations remain insufficient to clearly characterize current knowledge levels, training adequacy, and complication profiles among orthopedic surgeons.

In this context, the present study aims to evaluate contemporary knowledge levels, self-reported complication experiences, and training status related to pneumatic tourniquet use among orthopedic specialists and residents in our country.

## Materials and methods

This study was designed as a cross-sectional, descriptive survey aimed at evaluating the knowledge levels, self-reported clinical experiences, and training status related to pneumatic tourniquet use among orthopedic and traumatology specialists and resident physicians in Turkey. Orthopedic surgeons and residents working in public hospitals, private hospitals, university hospitals, and training and research hospitals across the country were invited to participate on a voluntary basis. No personal identifying information was collected, and anonymity was strictly maintained.

### Questionnaire design

The questionnaire was developed based on the principles of safe pneumatic tourniquet use outlined in the Association of periOperative Registered Nurses (AORN) 2007 guideline [14, 15]. The survey consisted of 19 items addressing tourniquet placement, pressure determination, duration of use, complication awareness, neurovascular assessment practices, and management during special conditions such as intravenous regional anesthesia (IVRA). Additional items evaluated participant demographics, clinical experience, prior training exposure, sources of knowledge acquisition, and self-reported complications (Table 1).

Although the questionnaire content was grounded in established guideline recommendations, formal psychometric validation procedures (including assessment of content validity, construct validity, test–retest reliability, and internal consistency) were not performed. Therefore, the instrument was used as an exploratory tool to assess general knowledge patterns and clinical practices.

### Data collection

The survey was administered online using the Google Forms platform and distributed through professional communication networks and social media channels. Data collection was conducted between April 1, 2025, and May 1, 2025. To prevent duplicate entries, participants were restricted to a single response. Participation was entirely voluntary. Given the online and voluntary nature of recruitment, a degree of selection bias is unavoidable, as individuals with greater interest in the topic may have been more likely to participate.

### Ethical approval

Ethical approval for the study was obtained from the XX University Ethics Committee. Electronic informed consent was obtained from all participants prior to survey initiation.

### Statistical analysis

Statistical analysis was performed using SPSS version 25.0 (IBM Corp., Armonk, NY, USA). Descriptive statistics were reported as frequencies and percentages for categorical variables. Group comparisons of categorical variables were performed using the Pearson chi-square test. A p-value of < 0.05 was considered statistically significant. Due to the cross-sectional design and sample structure, multivariable regression analysis and adjustment for potential confounders were not performed. Effect size calculations were also not included. These limitations were considered in the interpretation of the results.

### Self-reported data and bias considerations

All complication data were based solely on participants’ self-reported experiences and were therefore subject to recall bias and reporting bias. No independent medical record verification was performed.

**Table 1:** Survey questions

*1. How many years of experience do you have in orthopedics?* (0–2 years [Resident]; 3–5 years [Resident]; 6–10 years [Specialist]; >10 years [Senior Specialist]) *2. What is the type of institution you work in?* (University hospital; Training and research hospital; State hospital; Private hospital; Other) *3. Have you received any training on pneumatic tourniquet use?* (Yes, both theoretical and practical; Yes, theoretical only; No) *4. How do you obtain your knowledge about pneumatic tourniquet use?* (Medical school education; Experience during residency; Specialist training courses; Academic articles and guidelines; Learning from colleagues; Manufacturer training programs) *5. How frequently do you use a tourniquet?* (In almost every surgery; Several times a week; Several times a month; Rarely; Never) *6. In which areas do you feel you lack knowledge regarding pneumatic tourniquet use?*( Methods for determining tourniquet pressure; Maximum safe duration; Prevention of complications; Maintenance/cleaning of devices; Details of tourniquet protocols) *7. Would you like to receive further training or attend courses?* (Yes, practical training; Yes, theoretical + practical training; No) *8. What complications do you most frequently encounter during tourniquet use?* (Skin injuries; Nerve injury; Muscle injury; Venous thromboembolism; Other) *9. Do you perform a neurovascular examination of the patient after tourniquet use?* Yes; No) *10. Which precautions should be taken to prevent injuries related to tourniquet use?* (Proper cuff width and placement; Adjusting pressure to patient; Not exceeding safe time; Evaluating limb after release) *11. What is the most important factor when selecting a tourniquet cuff?* (Size/shape of the limb; Cuff color; Brand; Year of manufacture) *12. How should the tourniquet cuff be placed on the limb?* (Wider part distally; Wider part proximally; Should be moved/slid; Exact fit not important) *13. What is the most suitable material for the tourniquet cuff?* (Plastic; Rubber; Soft, flexible, cleanable materials; Cotton fabric) *14. Which method should be used for exsanguination of the limb?* (Esmarch bandage; Elevation; Local anesthetic injection; Nothing) *15. What is the most dangerous error encountered during IVRA?* (Releasing the wrong cuff; Premature drug release; Inappropriate cuff selection; Not administering oxygen) *16. What is the most common complication encountered when tourniquet time is prolonged?* (Skin redness; Nerve injury; Increased muscle strength; Accelerated circulation) *17. How should complications related to tourniquet use be reported?* (Only to surgeon; After discharge; To hospital quality management system; Reporting not necessary) *18. Which physiological changes may be observed during tourniquet deflation?* (Decrease in blood pressure; Decrease in oxygen saturation; Increase in CO₂ levels; All) *19. How should tourniquet deflation be managed when tourniquets are used on both limbs?* (Deflate simultaneously; Deflate 30–45 min apart; Wait for pressure equalization; Timing not important)	

## Results

### Participant characteristics

A total of 300 orthopedic specialists and residents participated in the study. Most participants were senior specialists with ≥ 10 years of experience (*n* = 136, 45.3%). The largest proportion of participants worked in training and research hospitals (*n* = 129, 43.0%). Tourniquet use was highly prevalent, with 146 participants (48.7%) reporting use in nearly every surgery and 109 (36.3%) reporting use several times per week (Table 2).

**Table 2:** Professional experience, workplace and frequency of tourniquet use among participants

	*N*	%
Professional experience		
0–2 years	33	11.0
2–5 years	52	17.3
6–10 years	79	26.3
≥ 10 years	136	45.3
Workplace		
Training and Research Hospital	129	41.3
University Hospital	70	23.3
Private Hospital	53	17.7
State Hospital	39	13.0
Private Clinic	9	2.3
Frequency of tourniquet use		
In almost every surgery	146	48.7
Several times a week	109	36.3
Several times a month	23	7.7
Rarely	18	6.0

### Training status and perceived knowledge deficiencies

A total of 107 participants (35.7%) reported having received both theoretical and practical training on pneumatic tourniquet use, while 29 (9.7%) reported receiving only theoretical training. The remaining 164 participants (54.6%) reported no formal training. Regarding future educational needs, 169 participants (56.3%) expressed a desire for both theoretical and practical training, 40 (13.3%) requested only practical training, and 91 (30.3%) stated that their current knowledge was sufficient. The most frequently reported perceived deficiencies were insufficient knowledge of tourniquet usage protocols (56%), pressure determination methods (44%), and maximum safe duration of use (35%).

### Complications related to tourniquet use

The most frequently self-reported complications associated with pneumatic tourniquet use were skin injury (*n* = 124, 41.3%), muscle injury or weakness (*n* = 112, 37.3%), and nerve injury (*n* = 91, 30.3%), representing predominantly soft-tissue and neurocompressive complications. Vascular complications such as venous thromboembolism were reported by 36 participants (12.0%). Rare but serious complications included compartment syndrome (*n* = 1, 0.3%) and blister formation (*n* = 2, 0.7%) (Table 3). Following tourniquet release, 268 participants (89.3%) reported routinely performing a post-tourniquet neurovascular examination.

**Table 3:** Complications experienced during the use of pneumatic tourniquets

	*N*	%
Skin injury	124	41
Muscle injury	112	37
Nerve injury	91	30
No complications	44	14
Venous thromboembolism	36	12
Pain	11	3
Other	9	2

### Overall knowledge responses

Regarding preventive measures against tourniquet-related injury, 255 participants (85.0%) selected all correct options. Nearly all participants correctly identified the most important factor in cuff selection (*n* = 291, 97.0%). Correct responses were also frequent for cuff placement method (74%), suitable cuff material (74%), and exsanguination technique (64%) (Supplementary Table 1).

For IVRA-related knowledge, only 72 participants (24.0%) correctly identified early anesthetic release as the most dangerous error, while 34% incorrectly selected cuff release as the primary error. Correct identification of the most common complication associated with prolonged tourniquet use was observed in 46.7% of the participants. Correct knowledge regarding physiological changes during deflation was present in 68.2%, and correct bilateral deflation management in 59.3% (Supplementary Table 2).

### Comparison by institution type

Correct identification of the most common complication resulting from prolonged tourniquet duration differed significantly according to institution type. Participants working in university and training-research hospitals demonstrated a correct response rate of 43.1%, whereas participants working in other institutions demonstrated a higher correct response rate of 58.1% (*p* = 0.013). No statistically significant differences were observed for other survey items based on institution type.

### Comparison by professional experience

Participants were grouped according to experience as 0–2 years, 2–5 years, 6–10 years, and ≥ 10 years. The correct response rate for the most common complication of prolonged tourniquet use was 42.4% in the 0–2 year group, decreased to 30.8% in the 2–5 year group, and increased to 46.8% in the 6–10 year group and 57.4% in the ≥ 10 year group, demonstrating a statistically significant difference among experience groups (*p* = 0.010). No significant differences were observed for other survey items (Table 4).^1^

### Comparison by training status

Participants were categorized into three groups based on training status: theoretical and practical training, only theoretical training, and no training. A significant difference was observed only for the IVRA-related safety question, where correct response rates paradoxically increased from the trained group (15.0%) to the untrained group (29.3%) (*p* = 0.024). No other knowledge items differed significantly between training groups (Table 5).^2^  

### Comparison by history of complications

When knowledge levels were compared according to whether participants had previously experienced tourniquet-related complications, no overall significant association was identified. However, participants who reported prior complications demonstrated a significantly higher correct response rate regarding proper reporting of tourniquet-related complications (*p* = 0.026). All other comparisons showed no statistically significant differences (Table 6).^3^

**Table 4:** Accuracy of survey responses according to experience

		0–2 years		2–5 years		6–10 years		Over 10 years		P
		N	%	N	%	N	%	N	%	
Precautions taken to prevent patient injuries related to tourniquet use	True	22	66.7	37	71.2	65	82.3	101	74.3	0.272
	False	11	33.3	15	28.8	14	17.7	35	25.7	
The most important factor when selecting a tourniquet cuff	True	33	100.0	50	96.2	78	98.7	130	95.6	0.408
	False	0	0.0	2	3.8	1	1.3	6	4.4	
The method of placing the tourniquet cuff on the limb	True	23	69.7	41	78.8	57	72.2	101	74.3	0.778
	False	10	30.3	11	21.2	22	27.8	35	25.7	
The most suitable material for the tourniquet cuff	True	22	66.7	40	76.9	64	81.0	96	70.6	0.262
	False	11	33.3	12	23.1	15	19.0	40	29.4	
Methods used for exsanguination	True	16	48.5	37	71.2	53	67.1	86	63.2	0.174
	False	17	51.5	15	28.8	26	32.9	50	36.8	
The most dangerous mistake in IVRA	True	10	30.3	14	26.9	21	26.6	27	19.9	0.469
	False	23	69.7	38	73.1	58	73.4	109	80.1	
The most common complication resulting from prolonged tourniquet duration	True	14	42.4	16	30.8	37	46.8	78	57.4	**0.010**
	False	19	57.6	36	69.2	42	53.2	58	42.6	
Reporting of complications related to tourniquet use.	True	22	66.7	29	55.8	52	65.8	89	65.4	0.600
	False	11	33.3	23	44.2	27	34.2	47	34.6	
Physiological changes observed during tourniquet deflation	True	29	87.9	33	63.5	52	65.8	90	66.2	0.076
	False	4	12.1	19	36.5	27	34.2	46	33.8	
Management of tourniquet deflation in bilateral tourniquet use	True	17	51.5	28	53.8	45	57.0	88	64.7	0.384
	False	16	48,5	24	46,2	34	43,0	48	35,3	

**Table 5:** Accuracy of survey responses according to educational status

		Theoretical and practical		Only practical		No training		P
		N	%	N	%	N	%	
Precautions taken to prevent patient injuries related to tourniquet use	True	81	75.7	23	79.3	121	73.8	0.8
	False	26	24.3	6	20.7	43	26.2	
The most important factor when selecting a tourniquet cuff	True	103	96.3	28	96.6	160	97.6	0.82
	False	4	3.7	1	3.4	4	2.4	
The method of placing the tourniquet cuff on the limb	True	80	74.8	20	69.0	122	74.4	0.807
	False	27	25.2	9	31.0	42	25.6	
The most suitable material for the tourniquet cuff	True	81	75.7	21	72.4	120	73.2	0.879
	False	26	24.3	8	27.6	44	26.8	
Methods used for exsanguination	True	65	60.7	22	75.9	105	64.0	0.323
	False	42	39.3	7	24.1	59	36.0	
The most dangerous mistake in IVRA	True	16	15.0	8	27.6	48	29.3	**0.024**
	False	91	85.0	21	72.4	116	70.7	
The most common complication resulting from prolonged tourniquet duration	True	55	51.4	17	58.6	73	44.5	0.274
	False	52	48.6	12	41.4	91	55.5	
Reporting of complications related to tourniquet use.	True	74	69.2	18	62.1	100	61.0	0.38
	False	33	30.8	11	37.9	64	39.0	
Physiological changes observed during tourniquet deflation	True	75	70.1	20	69.0	109	66.5	0.816
	False	32	29.9	9	31.0	55	33.5	
Management of tourniquet deflation in bilateral tourniquet use	True	70	65.4	15	51.7	93	56.7	0.246
	False	37	34.6	14	48.3	71	43.3	

**Table 6:** Accuracy of survey responses according to whether complications were experienced during pneumatic tourniquet use

		Yes		No		P
		N	%	N	%	
Precautions taken to prevent patient injuries related to tourniquet use	True	191	74.3	34	79.1	0.505
	False	66	25.7	9	20.9	
The most important factor when selecting a tourniquet cuff	True	248	96.5	43	100.0	0.213
	False	9	3.5	0	0.0	
The method of placing the tourniquet cuff on the limb	True	189	73.5	33	76.7	0.658
	False	68	26.5	10	23.3	
The most suitable material for the tourniquet cuff	True	190	73.9	32	74.4	0.946
	False	67	26.1	11	25.6	
Methods used for exsanguination	True	170	66.1	22	51.2	0.058
	False	87	33.9	21	48.8	
The most dangerous mistake in IVRA	True	62	24.1	10	23.3	0.902
	False	195	75.9	33	76.7	
The most common complication resulting from prolonged tourniquet duration	True	126	49.0	19	44.2	0.557
	False	131	51.0	24	55.8	
Reporting of complications related to tourniquet use	True	158	61.5	34	79.1	**0.026**
	False	99	38.5	9	20.9	
Physiological changes observed during tourniquet deflation	True	174	67.7	30	69.8	0.788
	False	83	32.3	13	30.2	
Management of tourniquet deflation in bilateral tourniquet use	True	153	59.5	25	58.1	0.863
	False	104	40.5	18	41.9	

## Discussion

This study demonstrates that although pneumatic tourniquets are widely used in orthopedic and traumatology practice, substantial gaps persist in theoretical knowledge, structured training, and complication awareness. These deficiencies, observed across different experience levels, suggest that current clinical exposure alone may be insufficient to ensure safe and standardized tourniquet application.

Although senior specialists constituted nearly half of the study population, substantial knowledge gaps remained evident even within this experienced group. This finding indicates that professional experience, while valuable, cannot substitute for structured, guideline-based education in ensuring safe and standardized pneumatic tourniquet use. In line with this observation, most participants reported acquiring their knowledge primarily through informal clinical exposure during residency rather than through formal educational programs. Moreover, prior exposure to tourniquet-related complications did not consistently improve overall knowledge performance, although it modestly increased awareness regarding proper complication reporting. Together, these findings underscore that experiential learning alone may not systematically improve patient-safety-oriented knowledge. Similar patterns have been reported internationally. Albaker et al. demonstrated that among orthopedic surgeons in Saudi Arabia, significant deficiencies persisted across multiple core domains of tourniquet knowledge—including limb occlusion pressure, contraindications, and documentation standards—despite varying levels of clinical experience. Notably, the incidence of post-tourniquet syndrome and neurovascular complications was not significantly associated with surgeon expertise level, reinforcing the concept that clinical seniority alone does not guarantee safe and guideline-adherent tourniquet practice [16]. 

The low rate of formal training identified in this study is consistent with previous national reports. Yalçınkaya et al. demonstrated that a considerable proportion of orthopedic surgeons applied tourniquets at cuff pressures well above the limits recommended in the literature and relied predominantly on senior colleagues’ instructions rather than evidence-based sources when determining tourniquet pressure and inflation time. Moreover, despite many participants stating that they adhered to the literature, only a small minority applied pressures within safe limits, revealing substantial misinterpretation of scientific recommendations [12]. Similarly, Boya et al. reported that nearly 70% of orthopedic clinics in Turkey lacked any form of periodic formal education on tourniquet use, that assistant staff and operating room personnel frequently applied the tourniquet, and that scientifically valid low-pressure strategies such as limb occlusion pressure–based adjustment were almost entirely absent in routine practice. Furthermore, wide variability in pressure settings, inflation times, and responses to prolonged tourniquet use was observed, underscoring unsafe heterogeneity in daily practice [13]. 

The relatively high rates of self-reported tourniquet-related complications observed in the present study contrast strikingly with contemporary international data. A large national population-based survey from Norway demonstrated that tourniquet-related nerve injury is currently a very rare complication when modern pneumatic tourniquet systems are used in accordance with standardized cuff pressures, controlled ischemia duration, and regulated reperfusion protocols. This pronounced difference suggests that variation in clinical practice patterns, adherence to guidelines, and institutional safety frameworks plays a pivotal role in determining complication profiles [17]. In contrast, a nationwide survey from Nigeria revealed widespread reliance on non-pneumatic tourniquets with indeterminate and potentially excessive pressures, frequent application by non-physician personnel, limited documentation of inflation–deflation times, and underutilization of controlled pneumatic systems, together with high rates of tourniquet palsy and permanent neurologic injury [18]. Collectively, these contrasting international data indicate that tourniquet-related complications are influenced not only by individual knowledge or experience, but also—perhaps more importantly—by broader systemic factors such as device availability, institutional protocols, staff training, and perioperative safety culture. Nevertheless, the findings of the present study should be interpreted with caution, as complication data were based solely on retrospective self-reporting and are therefore susceptible to recall bias. Overall, these observations support the view that tourniquet-related complications should be regarded as multifactorial rather than solely knowledge-driven events.

Participants demonstrated relatively good proficiency in basic technical aspects of tourniquet use, such as cuff selection, placement, material choice, and general preventive measures. These skills are likely acquired through routine clinical exposure. However, deficiencies became more pronounced in advanced safety-critical domains such as pressure individualization, IVRA-related risks, prolonged inflation complications, and systemic effects during deflation. This pattern suggests that while procedural habits may be learned through practice, deeper physiological and safety-focused knowledge requires structured theoretical reinforcement.

Individualized pressure adjustment based on limb occlusion pressure (LOP) is a central recommendation in current guidelines. While Kanchanathepsak et al. reported that standardized tourniquet pressures may be safely applied in selected short-duration upper-extremity procedures, current guideline consensus continues to favor individualized LOP-based pressure setting as the most reliable strategy for minimizing neurovascular injury [14, 15, 19, 20]. In the present study, nearly half of the participants reported feeling inadequate in determining appropriate tourniquet pressure, and a substantial proportion appeared unaware of patient-specific pressure adjustment principles, highlighting a persistent translational gap between guideline recommendations and routine clinical practice.

Knowledge regarding exsanguination techniques also showed important gaps. Although exsanguination improves the surgical field and allows lower cuff pressures, it is contraindicated in specific clinical scenarios such as malignant tumors and suspected infections [7, 21]. The finding that more than one-third of participants lacked full knowledge in this domain raises concerns regarding patient-specific risk assessment in daily practice.

Prolonged tourniquet inflation remains one of the most critical determinants of tourniquet-related morbidity. In a review study, Xacur-Trabulce et al. reported the most common complication due to prolonged tourniquet use in emergency departments as nerve damage [22]. Similarly, Dayan et al. found nerve damage as the most common complication due to prolonged tourniquet use in battlefield settings [23]. Although nerve injury was the correct response in our survey, the overall accuracy rate remained below 50%, highlighting a substantial knowledge gap involving a major preventable complication.

Awareness regarding life-threatening IVRA-related complications was particularly concerning. Early systemic release of local anesthetics during IVRA may result in severe cardiotoxicity and even cardiac arrest [24–26]. Despite the clinical importance of this complication, only one-quarter of participants identified it correctly, and paradoxically, the lowest accuracy rate was observed among those who reported prior training. This finding suggests potential deficiencies in the depth, quality, or retention of IVRA-related education within existing training programs.

Tourniquet deflation represents another physiologically critical phase. Sudden release may induce hypotension, hypoxemia, hypercapnia, metabolic acidosis, hyperkalemia, and myoglobin release, particularly during lower-extremity, bilateral, or prolonged applications [27–29]. The limited accuracy observed in knowledge related to these systemic effects and bilateral deflation management suggests suboptimal awareness of peri-deflation physiology and highlights the importance of close coordination between the surgical and anesthesia teams.

Several limitations must be considered when interpreting the present findings. First, the study relied on self-reported survey data, which are subject to recall bias and social desirability bias. Second, voluntary online participation introduces unavoidable selection bias. Third, the cross-sectional design does not permit causal inference between knowledge deficits and complication occurrence. Finally, the lack of formal psychometric validation of the questionnaire limits the precision of knowledge measurement. These factors collectively restrict the generalizability of the results. Taken together, the findings suggest that existing deficiencies in pneumatic tourniquet practice are not merely individual but reflect broader systemic gaps in structured education and institutional policy. Therefore, beyond generic calls for “more training,” targeted and implementable strategies are required. These include the establishment of standardized national training modules, mandatory simulation-based certification before independent tourniquet use, periodic competency reassessment, formal institutional tourniquet safety protocols, and interdisciplinary perioperative safety checklists. Such structured approaches may improve patient safety, reduce practice variability, and minimize preventable tourniquet-related complications.

## Supplementary Information

Below is the link to the electronic supplementary material.

Supplementary Material 1

Supplementary Material 2

## Data Availability

The survey dataset generated in this study is available from the corresponding author upon reasonable request.

## Footnotes

1 Bold values represent statistically significant results (p < 0.05).

2 Bold value represent statistically significant results (p < 0.05).

3 Bold value represent statistically significant results (p < 0.05).
