# Supplementary material for: Knowledge, training, and practice patterns in pneumatic tourniquet use among orthopedic physicians: a national cross-sectional survey
Source: Arch Orthop Trauma Surg. 2026 Feb 2;146(1):39. doi: 10.1007/s00402-025-06176-1 (PMC12864187; doi:10.1007/s00402-025-06176-1)
Supplement: Supplementary file 1 — Supplementary Material 1 [file 402_2025_6176_MOESM1_ESM.docx]

**Supplementary table 1.** Survey responses

| **Precautions taken to prevent patient injuries related to tourniquet use** | **N** | **%** |
| --- | --- | --- |
| Proper cuff width and placement; adjusting tourniquet pressure according to the patient; not exceeding the maximum tourniquet time; careful evaluation of the limb after tourniquet release | 225 | 75.0 |
| Proper cuff width and placement; adjusting tourniquet pressure according to the patient; not exceeding the maximum tourniquet time | 33 | 11.0 |
| Not exceeding the maximum tourniquet time | 9 | 3.0 |
| Proper cuff width and placement | 8 | 2.7 |
| Others | 25 | 8.3 |
| **The most important factor when selecting a tourniquet cuff** | **N** | **%** |
| Size and shape of the limb | 291 | 97.0 |
| Others | 9 | 3.0 |
| **The method of placing the tourniquet cuff on the limb** | **N** | **%** |
| With the wider part positioned proximally | 222 | 74.0 |
| With the wider part positioned distally | 29 | 9.7 |
| It should be moved/slid during application | 19 | 6.3 |
| Others | 30 | 10.0 |
| **The most suitable material for the tourniquet cuff** | **N** | **%** |
| Soft, flexible, and cleanable materials | 222 | 74.0 |
| Rubber | 25 | 8.3 |
| Soft, flexible, and cleanable materials; cotton fabric | 13 | 4.3 |
| Rubber; soft, flexible, and cleanable materials | 11 | 3.7 |
| Others | 29 | 2.7 |
| **Methods used for exsanguination** | **N** | **%** |
| An Esmarch bandage should be used; the limb should be elevated | 192 | 64.0 |
| An Esmarch bandage should be used | 71 | 23.7 |
| The limb should be elevated | 34 | 11.3 |
