# Supplementary material for: Knowledge, training, and practice patterns in pneumatic tourniquet use among orthopedic physicians: a national cross-sectional survey
Source: Arch Orthop Trauma Surg. 2026 Feb 2;146(1):39. doi: 10.1007/s00402-025-06176-1 (PMC12864187; doi:10.1007/s00402-025-06176-1)
Supplement: Supplementary file 2 — Supplementary Material 2 [file 402_2025_6176_MOESM2_ESM.docx]

**Supplementary table 2.** Survey responses (continued)

| **The most dangerous mistake in IVRA** | **N** | **%** |
| --- | --- | --- |
| Releasing the wrong cuff | 102 | 34.0 |
| Inappropriate cuff selection | 74 | 24.7 |
| Early release of the anesthetic drug | 72 | 24.0 |
| No response | 22 | 7.3 |
| Others | 30 | 10.0 |
| **The most common complication resulting from prolonged tourniquet duration** | **N** | **%** |
| Nerve injury | 145 | 48.3 |
| Skin redness | 129 | 43.0 |
| Skin redness and nerve injury | 26 | 8.6 |
| **Reporting of complications related to tourniquet use** | **N** | **%** |
| Should be recorded in the hospital quality management system | 192 | 64.0 |
| Should be noted after the patient is discharged | 43 | 14.3 |
| Should be reported only to the surgeon | 39 | 13.0 |
| Others | 26 | 2.7 |
| **Physiological changes observed during tourniquet deflation** | **N** | **%** |
| All | 206 | 68.7 |
| Decrease in blood pressure | 54 | 18.0 |
| Decrease in blood pressure and oxygen saturation | 13 | 4.3 |
| Others | 27 | 9.0 |
| **Management of tourniquet deflation when tourniquets are used on both limbs** | **N** | **%** |
| Tourniquets should be deflated at least 30–45 minutes apart | 178 | 59.3 |
| Wait until the pressure equalizes | 69 | 23.0 |
| Deflate at least 30–45 minutes apart **and** wait until the pressure equalizes | 18 | 6.0 |
| Others | 35 | 11.6 |
